# Chromosome-level genome assembly of the sap beetle *Glischrochilus (Librodor) japonius* (Coleoptera: Nitidulidae)

**DOI:** 10.1038/s41597-025-04774-7

**Published:** 2025-04-29

**Authors:** Panpan Li, Xinyuan Fan, Sheng Li, Yijie Tong, Zhehao Tian, Yingming Zhang, Shaolong Wu, Can Wang, Yansong Xiao, Guoquan Wang, Ming Bai

**Affiliations:** 1 Guangxi Key Laboratory of Agro-environment and Agric-products Safety, National Demonstration Center for Experimental Plant Science Education, College of Agriculture, Guangxi University, Nanning, Guangxi 530004 China; 2 Key Laboratory of Animal Biodiversity Conservation and Integrated Pest Management (Chinese Academy of Sciences), Institute of Zoology, Chinese Academy of Sciences, Beijing, 100101 China; 3 Key Laboratory of Ecology of Rare and Endangered Species and Environmental Protection, Guangxi Normal University, Ministry of Education, Guilin, Guangxi 541001 China; 4 School of Forestry and Prataculture, Ningxia University, Yinchuan, Ningxia 750021 China; 5 Guangdong Chebaling National Nature Reserve, Shaoguan, Guangdong 512500 China; 6 Tobacco Company of Hunan Province, Changsha, Hunan China; 7 Chenzhou Tobacco Company of Hunan Province, Changsha, Hunan China; 8 College of Plant Protection, Hebei Agricultural University, Baoding, 071001 China; 9 Northeast Asia Biodiversity Research Center, Northeast Forestry University, Harbin, 150040 China; 10 Academy of Plateau Science and Sustainability, Qinghai Normal University, Xining, 810016 China

**Keywords:** Molecular biology, Zoology

## Abstract

Sap beetles are widely distributed in the Holarctic and tropical regions, with diverse feeding habits and strong adaptability. They play important roles in the decay of plants, the spread of fungi and bacteria, and the carbon and nitrogen cycles in agricultural ecosystems. Here, we provide an annotated, chromosome level reference genome assembly for a sap beetle *Glischrochilus (Librodor) japonius* (Motschulsky, 1857), a member of the family Nitidulidae, assembled using PacBio HiFi and Hi-C data from female specimens. The final assembly has a total size of 789.06 Mb, with 94.91% of the sequence successfully anchored to 10 chromosomes. The scaffold N50 is 77.84 Mb, and BUSCO (endopterygota_odb10 database) completeness is 97.20%. Repetitive elements comprise 54.67% of the genome (431.38 Mb). We identified 1,673 noncoding RNAs and predicted 22,526 protein-coding genes in the genome. This genome will serve as a valuable resource for advancing our understanding of the evolution and ecology of sap beetles, and will facilitate comparative studies of genome structure within the Nitidulidae family.

## Background & Summary

The group of sap beetles (Coleoptera: Nitidulidae) is recognized as the largest family in Cucujoidea, comprises approximately 350 genera and at least 4,500 species^1^. This family has a widespread distribution, predominantly across the Holarctic and tropical regions, while certain subfamilies, such as Calonecrinae and Maynipeplinae, are localized to Southeast Asia and Africa, respectively. Sap beetles are found in nearly all major biogeographic regions and occupy a wide range of ecological niches^2,3^. Members of the Nitidulidae family are characterized by one of the most varied diets among all beetle families. The larvae typically inhabit areas near sap and primarily feed on it. In contrast, adult beetles exhibit a diverse range of feeding behaviors, including consumption of flowers, fruits, fungi, stored food products, decaying or fermenting plant materials, carrion, and even other insects^4,5^. Their high adaptability and widespread distribution allow them to thrive in various agricultural ecosystems. During feeding, sap beetles often introduce fungi and bacteria into damaged plant tissues, contributing to the subsequent decay and fermentation of plant and fruit matter^6–9^. Through these feeding habits, they play significant roles in the carbon and nitrogen cycles, positioning sap beetles as key indicator species for assessing the health and dynamics of forest ecosystems^10^.

Recent analyses of both morphological and molecular data provide strong support for the monophyly of Nitidulidae^2,10^. Phylogenetic analysis have suggested that the families Kateretidae and Smicripidae are sister groups to Nitidulidae, collectively forming the informal nitidulid series^11,12^. Nitidulids represent a relatively old lineage within Cucujoidea that has been recorded from the Mesozoic^13,14^. Based on molecular clock analyses, the possible origin of Nitidulidae can be dated back to the Early Cretaceous^15^, although a calibrated phylogeny of Coleoptera date back into the Late Jurassic, around 160 Mya^16^. Research on the molecular evolution of Nitidulidae has predominantly focused on taxonomic classification and phylogenetic relationships, using multi-locus DNA sequence data^2,11^ and mitochondrial genome data^10,17^. However, only a few studies have focused on the genus level^18^. Due to the lack of large-scale genomic data for Nitidulidae, the biogeographic history and the genetic mechanisms underlying their development and adaptation remain unresolved.

*Glischrochilus* Reitter, 1873 is a genus within the subfamily Cryptarchinae, comprising over 40 recognized species primarily found in the Holarctic Region, with some extending into the Oriental Region. China has the highest diversity of *Glischrochilus* species^11,19^. *Glischrochilus (Librodor) japonius* (Motschulsky, 1857) is small in size, with a body length of 7–14 mm, and has an oval shape. It has remarkable sexual dimorphism in the mandibles, strongly developed asymmetrical mandible in males, but moderately developed in females^20^. Nitidulidae is currently represented by only two genomes in NCBI (assessed: Nov. 27, 2024): *Aethina tumida* Murray, 1867 (assembly ID: GCA_024364675.1, GCA_024364635.1, GCA_001937115.1)^21^ and *Brassicogethes aeneus* (Fabricius, 1775) (assembly ID: GCA_921294245.1)^22^, both of which has been assembled to the chromosome level.

In this study, we used Illumina, HIFI, and Hi-C sequencing technologies to present the successful assembly of the chromosome-level reference genome of *Glischrochilus (Librodor) japonius*. The final assembly has a total size of 789.06 Mb, with 94.91% of the sequence successfully anchored to 10 chromosomes. This represents the first chromosome-level genome for the genus will enhance our understanding of sap beetle distribution and evolution. It will assist in comparative studies of genome structure and phylogenetic relationships within the family Nitidulidae.

## Methods

### Sample preparation

Adult specimens of *G. japonius* were collected from Huaiyuan County, Bengbu City, Anhui Province, China, during April and May 2024. A total of thirteen adult female *G. japonius*, with guts and mouthparts removed to minimize potential microbial contamination, were rapidly frozen in liquid nitrogen, stored at −80 °C, prepared for genome sequencing.

### Genomic DNA sequencing

A single adult female *G. japonius* specimen was used for Illumina sequencing, utilizing the TGuide Smart Universal DNA Kit (Tiangen) for DNA extraction. The DNA library for Illumina sequencing was constructed by Berry Genomics Company (Beijing, China) and sequenced on the Illumina NovaSeq X Plus platform. After filtering out adapter sequences and low-quality reads using Fastp (version 0.23.4)^23^, short-read sequencing generated 35.31 Gb (49 × coverage) of clean data (Table 1).

**Table 1:** The clean sequencing data and strategy used in genome assembly of *G. japonius*.

Sequencing strategy	Illumina	PacBio	Hi-C	RNA-seq
Clean data (Gb)	35.31	31.22	121.00	6.70
Coverage (×)	~49	~40	~153	—
N50 (bp)	150	15161	150	150
GC content (%)	31.28	31.28	33.34	39.39
Q20 (%)	98.17	—	98.54	97.74
Q30 (%)	94.91	—	95.75	97.74

Four adult female *G. japonius* specimens were used for PacBio HiFi whole genome sequencing, seven adult female *G. japonius* specimens were used for Hi-C sequencing. Genomic DNA extraction and library construction were completed by Novogene Company (Beijing, China), then PacBio HiFi and Hi-C were sequenced on PacBio Revio platform and Illumina Novaseq X plus platform. After filtering out adapter sequences and low-quality reads using Fastp (version 0.23.4)^23^, PacBio sequencing generated nearly 31.22 Gb (40 × coverage) HiFi clean data, Hi-C sequencing generated 121.00 Gb clean data (153 × coverage) (Table 1).

### Transcriptome sequencing

Total RNA was extracted from a single adult female *G. japonius* using the Trizol reagent protocol. RNA-seq libraries for conventional transcriptome sequencing were constructed by Novogene (Beijing, China) and sequenced on the Illumina NovaSeq X Plus platform. After filtering out adapter sequences and low-quality reads using Fastp (version 0.23.4)^23^, transcriptome sequencing generated nearly 6.70 Gb clean data (Table 1).

### Genome characteristics estimation

**Fig. 1:**
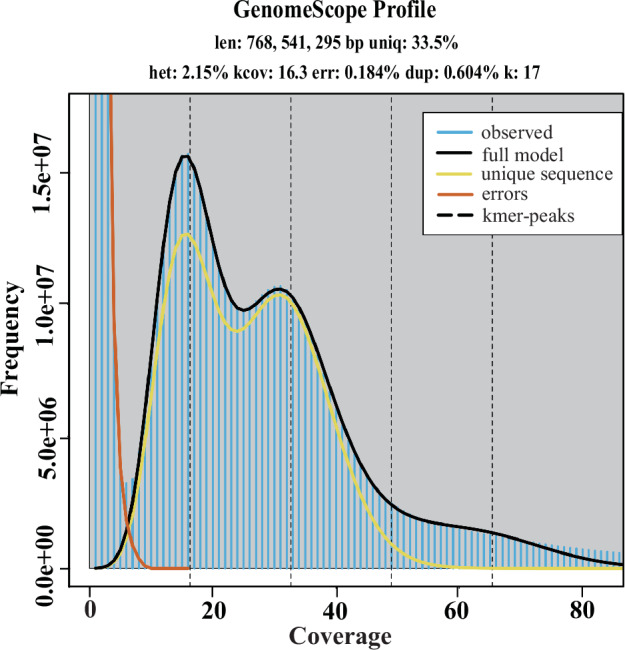
The K-mer (K = 17) distribution by GenomeScope.

The genome size of *G. japonius* was estimated by the k-mer method^24^ using Illumina clean data. K-mer distribution was estimated by using Jellyfish (version 2.3.1)^25^ with parameters -m 17 -C. The heterozygosity ratio was estimated by GenomeScope (version 2.0)^26^. Finally, the genome size was calculated according to the formula that Genome Size = K-mer coverage/Mean k-mer depth and the result showed that genome size is 734.36 Mb, heterozygosity is 2.15%, non-repetitive rate is 33.5% (Fig. 1).

### Genome assembly

For the genome assembly of *G. japonius*, HiFiasm (version 0.19.8-r603)^27^ was employed with default settings. To enhance continuity, purge_dups (version 1.2.6)^28^ was used to remove duplicated regions. The resulting draft genome had a total size of 778.19 Mb, consisting of 317 contigs with the N50 length of 6.60 Mb (Table S1).

**Fig. 2:**
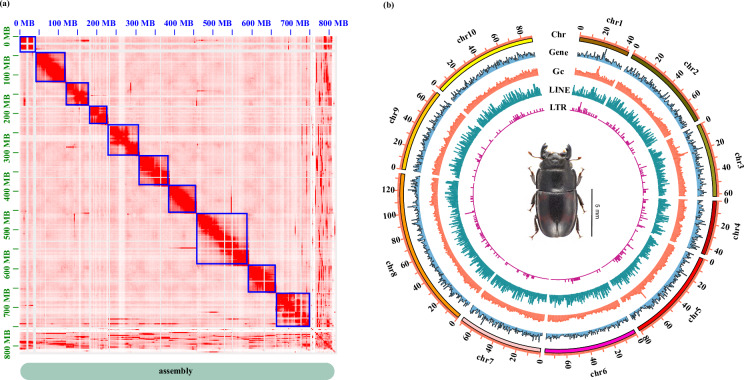
Landscape of the *G. japonius* genome. (**a**) The heatmap shows all-by-all interactions among 10 chromosomes of *G. japonius*. (**b**) Blocks on the outmost circle represent all 10 chromosomes of *G. japonius*. Peak plots from outer to inner circles represent the length of each chromosome, protein-coding genes, the GC content of each chromosome, the density of repeat sequences (LINE, long interspersed elements; LTR, long terminal repeat elements), respectively. Window size = 500 kb.

The Hi-C clean data was aligned to the draft genome using BWA (version 0.7.18)^29^, and Juicer (version 1.6, https://github.com/aidenlab/juicer) was used to identify valid paired-end sequences. Final genome assembly refinement was conducted using 3D-DNA^30^, ALLHIC (version 0.9.13)^31^ and Juicebox (https://github.com/aidenlab/juicebox), which enabled visualization and manual correction of the genome assembly. The length of the chromosome-level genome of *G. japonius* was 789.06 Mb, with a scaffold N50 of 77.84 Mb and 31.10% GC content (Table 2). The final assembly consists of 10 chromosomes, which are clearly distinguished from each other based on the chromatin interaction heatmap (Fig. 2a). The completeness of the genome was evaluated using BUSCO (5.3.2)^32^ with the endopterygota_odb10 database, which identified 97.20% of universally conserved genes, with 87.80% being single-copy genes and 9.40% represented as duplicates (Table 2). These results indicate that the anchored genome assembly is highly complete and of sufficiently high quality for subsequent detailed analyses.

**Table 2:** Statistics of Chromosome-level genome assembly of *G. japonius*.

Statistics	Value
Assembly size (Mb)	789.06
Number of scaffolds/contigs	666/3053
Longest scaffolds/contigs length (Mb)	134.04/6.15
scaffolds/contigs N50 (Mb)	77.84/1.375
scaffolds/contigs N90 (Mb)	42.70/0.11
GC content (%)	31.10
Repeat masker (%)	54.67
Number of assembled chromosomes	10
Number of genes	22,526
BUSCO (%)**-**scaffolds	
Complete	97.20
Complete single copy	87.80
Complete duplicated	9.40
Fragmented	0.90
Missing	1.90

### Repeat elements prediction

De novo prediction methods were used to detect repetitive elements. We employed RepeatModeler (version 2.0.5)^33^ to create a *de novo* repeat library. Long terminal repeat (LTR) retrotransposons were specifically identified using LTR_retriever (version 2.9.4)^34^. Tandem repeats were identified using Tandem Repeats Finder (version 4.09)^35^. Finally, RepeatMasker (version 4.0.9)^36^, based on the RepBase database^37^, was used to identify and annotate repetitive elements in the genome. The results indicated that repetitive sequences comprised 54.67% of the assembled genome of *G. japonius* (Table 3).

**Table 3:** Classification of repetitive sequences in *G. japonius* genome.

Category	Number of elements	Ratio (%) in genome
SINEs	0	0
LINEs	167,403	9.32
LTR elements	49,210	2.80
DNA transposons	219,420	8.30
unclassified	1,350,841	34.05
Small RNA	768	0.02

### Gene prediction

**Fig. 3:**
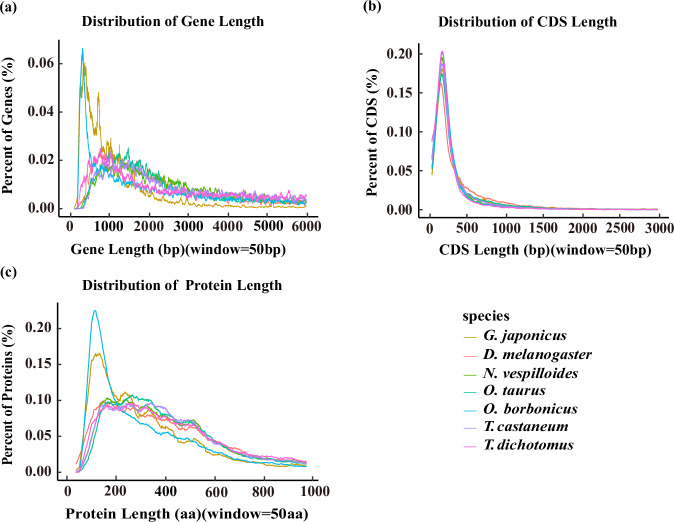
Distribution statistics of gene features among the seven species. The comparison of (**a**) Gene length, (**b**) CDS length, (**c**) Protein length in *G. japonius* and other six species.

Gene prediction in the assembled genome of *G. japonius* was conducted using a combination of homology, transcriptome data, and *ab initio* methods with *Tribolium castaneum* (GCA_031307605.1), *Cetonia aurata* (GCA_949128085.1), *Popillia japonica* (GCA_042919845.1), *Onthophagus taurus* (GCA_000648695.2), *Holotrichia oblita* (GCA_023690525.1), which were downloaded from NCBI (https://www.ncbi.nlm.nih.gov/). RNA-seq data were first aligned to the assembled genome using HISAT2 (version 2.2.1)^38^. Braker (version 3.0.8)^39–42^ integrates RNA-seq, protein evidence, and *ab initio* predictions. Initially, GeneMark-ETP (version 1.02)^40^ incorporated RNA-seq and protein alignment data to identify gene structures. These data were then used to train AUGUSTUS (version 3.5.0)^40^ specifically for *G. japonius*, refining its recognition of coding regions. Finally, AUGUSTUS (version 3.5.0)^40^ conducted genome-wide predictions in parallel, leveraging both extrinsic hints and *ab initio* modeling, resulting in the identification of 22,526 high-confidence genes (Table 2). The average mRNA length was 8,534.8 bp, and the average CDS length was 1,102.1 bp. We compared the different gene features of *G. japonius* with those of six species with high-quality assemblies: *Tribolium castaneum*, *Onthophagus taurus*, *Trypoxylus dichotomus*, *Oryctes borbonicus*, *Nicrophorus vespilloides*, and *Drosophila melanogaster*, which were downloaded from InsectBase2.0 (http://v2.insect-genome.com/) (Fig. 3).

### Gene function annotation

Protein-coding genes were aligned against various databases. Gene functional annotation proceeded by searching the UniProt protein sequence database (UniRef90)^43^ and Non-Redundant Protein Sequence database (NR) at the National Center for Biotechnology (NCBI) using Diamond (version 2.0.8.146)^42^. Pathway and ontology annotations were assigned using the Kyoto Encyclopedia of Genes and Genomes (KEGG)^44^, Gene Ontology (GO)^45,46^ and evolutionary genealogy of genes: non-supervised orthologous groups (eggNOG)^47^ by eggNOG-mapper (version 2.0.1)^48^. The protein families database (Pfam)^49^ was assigned using HMMER (version 2.2 g)^50^ and the protein domains were assigned using InterProScan (version 5.70-102.0)^51^ with InterPro database. Circos (version 0.69-8)^52^ was used to produce the visual diagram of *G. japonius* genomic characteristics (LTR, LINE, GENE, GC, and Chr) (Fig. 2b). Additionally, 21,932 genes (97.36%) have been annotated in at least one database (Table 4).

**Table 4:** The statistics of gene function prediction in *G. japonius* genome.

Functional database	Number	Percentage (%)
Annotated	21,932	97.36
Nr	21,931	97.32
Uniref90	21,923	97.32
eggnog	20,886	92.71
Interproscan	20,013	88.84
Pfam A	14,842	65.87
GO	9,393	41.69
KEGG	6,640	29.47

### Non-coding RNA annotation

For the annotation of non-coding RNAs (ncRNA), the draft genome was analyzed as follows: Transfer RNA (tRNA) genes were annotated using tRNAscan-SE (version 2.0.12)^53^ based on tRNA structural characteristics. Ribosomal RNA (rRNA) genes were predicted with RNAmmer (version 1.2)^54^. small nucleolar RNA (snRNA) and microRNA (miRNA) were predicted using Infernal (version 1.1.5)^55^. Collectively, these analyses revealed a total of 1,316 tRNA genes, 109 rRNA genes, 182 snRNA genes, and 66 miRNA genes (Table 5).

**Table 5:** The statistics of ncRNA gene prediction in *G. japonius* genome.

Category		Number	Total length (bp)	Average length	% of genome
tRNA		1316	101936	77.46	0.05842138
rRNA	18S	22	42215	1918.86	0.00097665
	28S	22	125547	5706.68	0.00097665
	5.8S	65	170143	2617.68	0.00288555
snRNA	CD-box	27	3275	121.30	0.00119861
	HACA-box	3	411	137	0.00013318
	splicing	152	21184	139.37	0.00674776
miRNA		66	5026	76.15	0.00292995

## Data Records

The Illumina, PacBio, and Hi-C sequencing data used for the genome assembly have been deposited at the National Center for Biotechnology Information (NCBI). The PacBio, Illumina, Hi-C, and transcriptome data can be found under identification numbers SRR31821647^56^, SRR31821641^57^, SRR31831788^58^, SRR31821622^59^. The assembled genome has been deposited in the NCBI assembly with the accession number JBDIXK01000000057^60^. The annotation file has been deposited in figshare database^61^. All sequencing data, as well as the results of genome assembly and annotation, have also been deposited in the Science Data Bank^62–66^.

## Technical Validation

The quality and completeness of chromosome assembly were evaluated using three independent approaches. First, the consensus quality values (QV) was estimated using Merqury (version 1.3)^67^ by comparing k-mers in the assembly to those found in the Illumina sequence reads. The results revealed that the QV for chromosome assembly was 64.65, demonstrating a high level of assembly accuracy. Secondly, we calculated the mapping rate as a measure of assembly accuracy. The mapping rate for PacBio and Illumina reads were 99.87% and 95.93%, which were calculated using Bedtools (version 3.31.1)^68^. The mapping rate for RNA reads was 90.36%, which was calculated using HISAT2(version 2.2.1)^38^. Thirdly, BUSCO (verson 5.3.2)^34^ was used to assess the completeness of the *G. japonius* genome assembly and genome annotation with the endopterygota_odb10 database. The completeness of the final genome assembly was 2063 (97.20%), containing 1864 (87.80%) single-copy BUSCOs, 199 (9.4%) duplicated BUSCOs, and 42 (1.9%) missing BUSCOs (Table 2). The analysis indicated that 90.30% of conserved orthologous genes were complete in the predicted protein-coding genes, comprising 81.40% single-copy and 8.90% duplicated genes (Table 6). The assessment results indicated that *G. japonius* genome assembly was complete, accurate, and of high quality.

**Table 6:** BUSCO analysis results of the *G. japonius* protein.

BUSCO (%)-Protein	
Complete	90.30
Complete single copy	81.40
Complete duplicated	8.90
Fragmented	1.50
Missing	8.20

## Supplementary information

Table S1. Statistics of draft genome assembly of G. japonius

## Data Availability

Programs used in data processing were executed with default parameters unless otherwise specified in the Methods section. No custom code was employed for these analyses.
