# Supplementary material for: Chromosome-level genome assembly of the sap beetle Glischrochilus (Librodor) japonius (Coleoptera: Nitidulidae)
Source: Sci Data. 2025 Apr 29;12:711. doi: 10.1038/s41597-025-04774-7 (PMC12041576; doi:10.1038/s41597-025-04774-7)
Supplement: Supplementary file 1 — Table S1. Statistics of draft genome assembly of G. japonius [file 41597_2025_4774_MOESM1_ESM.pdf]

Table of contents

Supplementary Tables	
Table S1. Statistics of draft genome assembly of *G. japonius*.	Page 2

Supplementary Table S1. Statistics of draft genome assembly of *G. japonius*.

Statistics	Value
Assembly size (Mb)	778.19
Number of scaffolds	300
Number of contigs	317
Longest scaffold length (Mb)	22.57
Longest contigs length (Mb)	22.57
Contig N50 (Mb)	6.60
Scaffolds N50 (Mb)	6.60
Contig N90 (Mb)	1.57
Scaffolds N90 (Mb)	1.62
GC content (%)	31.10
